# Cardiac Rehabilitation in Patients with Coronary Heart Disease – Provision, Attendance, and Outcomes: Results from the INTERASPIRE Survey from Fourteen Countries Across Six WHO Regions

**DOI:** 10.5334/gh.1458

**Published:** 2025-09-02

**Authors:** Kornelia Kotseva, Dirk De Bacquer, Catriona Jennings, John William McEvoy, Lars Ryden, Kausik K. Ray, Gregory Y. H. Lip, Iris Erlund, Sandra Ganly, Terhi Vihervaara, Agnieszka Adamska, Ana Abreu, Wael Almahmeed, Ade Meidian Ambari, Susan Connolly, Junbo Ge, Irene Gibson, Hosam Hasan-Ali, Sue Hennessy, Yong Huo, Piotr Jankowski, Rodney M. Jimenez, Jennifer Jones, Yong Li, Ahmad Syadi Mahmood Zuhdi, Abel Makubi, Amam Chinyere Mbakwem, Lilian Mbau, Jose Luis Navarro Estrada, Okechukwu Samuel Ogah, Elijah Nyainda Ogola, Adalberto Quintero–Baiz, Mahmoud Umar Sani, Maria Ines Sosa Liprandi, Jack Wei Chieh Tan, David R. Thompson, Miguel Alberto Urina Triana, Tee Joo Yeo, David Wood, Guy G. De Backer, on behalf of the INTERASPIRE Investigators

**Affiliations:** 1 University of Galway School of Medicine and National Institute for Prevention and Cardiovascular Health, Galway, Ireland; 2 Imperial College Healthcare NHS Trust, London, UK; 3 Department of Public Health and Primary Care, Ghent University, Belgium; 4 Department of Medicine Solna, Karolinska Institutet, Stockholm, Sweden; 5 Department of Public Health and Primary Care, Imperial College London, UK; 6 Liverpool Centre for Cardiovascular Science at University of Liverpool, Liverpool John Moores University and Liverpool Heart & Chest Hospital, Liverpool, UK; 7 Department of Clinical Medicine, Aalborg University, Aalborg, Denmark; 8 Medical University of Bialystok, Bialystok, Poland; 9 Biomarkers, Department of Government Services, Finnish Institute for Health and Welfare, Finland; 10 Cardiology Service, Cardiovascular Rehabilitation Unit, Hospital S. Maria (ULSSM), CAML, Lisbon, Portugal; 11 Faculty of Medicine University of Lisbon, ISAMB, IMPSP, CCUL, Lisbon, Portugal; 12 Cleveland Clinic Abu Dhabi, Al Mariah Island, Abu Dhabi, United Arab Emirates; 13 National Cardiovascular Center Harapan Kita, Department of Cardiology and Vascular Medicine, Faculty of Medicine, Universitas Indonesia, West Jakarta, Jakarta, Indonesia; 14 Fudan University Zhongshan Hospital, Shanghai, China; 15 Cardiovascular Medicine Department, Assiut University Heart Hospital, Faculty of Medicine, Assiut University, Assiut, Egypt; 16 Peking University First Hospital, Department of Cardiology, Institute of Cardiovascular Disease, Beijing, China; 17 Department of Internal Medicine and Geriatric Cardiology, Centre of Postgraduate Medical Education, Warsaw, Poland; 18 Philippine Heart Association & St. Luke’s Medical Center, Global City, Rizal Avenue Corner 32nd Street, Taguig City, Metro Manila, Philippines; 19 Department of Internal Medicine (Cardiology), Fudan University Huashan Hospital, 12 Wulumuqi Zhong Rd, Shanghai, China PR; 20 University Malaya Medical Centre, Cardiology Unit, Department of Medicine, Kuala Lumpur, Malaysia; 21 Muhimbili University of Health and Allied sciences (MUHAS), Dar es salaam, Tanzania; 22 College of Medicine, University of Lagos, Idi Araba, Lagos, Nigeria; 23 Kenya Cardiac Society, Nairobi, Kenya; 24 Italian Hospital of Buenos Aires, Gascon Buenos Aires, Argentina; 25 Cardiology Unit, Department of Medicine, University of Ibadan & University College Hospital, Ibadan, Nigeria; 26 Department of clinical medicine and therapeutics, University of Nairobi, Nairobi, Kenya; 27 Faculty of Health Sciences, University Simon Bolivar, Barranquilla, Colombia; 28 Department of Medicine, Bayero University Kano & Aminu Kano Teaching Hospital, Kano State, Nigeria; 29 Department of Cardiology, University Hospital Sanatorio Güemes, Buenos Aires, Argentina; 30 National Heart Centre Singapore, Singapore, Singapore; 31 Department of Cardiology, Duke-NUS Medical School, Singapore, Singapore; 32 Department of Cardiology, Sengkang General Hospital, Singapore, Singapore; 33 School of Nursing and Midwifery, Queen’s University Belfast, Belfast, UK; 34 Faculty of Health Sciences, Universidad Simon Bolivar, Barranquilla, Colombia; 35 National University Heart Centre Singapore and National University Hospital, National University Health System (NUHS) Tower Block, Cardiac Department, Singapore; 36 National Heart and Lung Institute, Imperial College London, London, UK

**Keywords:** cardiac rehabilitation, INTERASPIRE, coronary heart disease, secondary prevention, referral

## Abstract

**Background:**

INTERASPIRE was an observational study of patients with coronary heart disease (CHD) from 88 hospitals in 14 countries across all six WHO regions. The objective was to describe the proportions of patients referred to and attending cardiac rehabilitation (CR) programmes and to compare lifestyle and risk factor target achievement according to participation in a CR programme.

**Methods:**

Patients 18–80 years of age, with a first or recurrent coronary hospitalisation (acute coronary syndrome and/or revascularisation procedure) were identified and invited to an interview and examination, between six months and two years after the index hospitalisation.

**Results:**

Overall, 4,548 (21.1% female) patients were interviewed a median of 1.05 (interquartile range 0.76–1.45) years after hospitalization. Of those patients, 34.4% reported having been advised to participate in a CR programme, though the percentage varied widely by country, from 4.0% in Kenya to 69.6% in Poland. Among patients advised to participate in CR, 57.1% participated in ≥50% of all sessions, 15.4% participated in <50% of the sessions, and 27.4% did not participate at all. Only 19.6% of all patients recruited to the study attended ≥50% of sessions. Content of programmes reported by patients also varied enormously between countries. Low education level, elective PCI, or unstable angina as recruiting events were associated with lower attendance rates. Attendance at ≥50% of all CR sessions was associated with a lower prevalence of persistent smoking and physical inactivity, better control of blood pressure and LDL-cholesterol, and a higher use of cardioprotective medications.

**Conclusions:**

INTERASPIRE provides a standardised international picture of CR provision and attendance in patients with CHD. Despite CR being a Class 1 recommendation in all international guidelines, only one third of CHD patients reported being advised to attend any form of CR and just one in five patients attended 50% of the sessions, with striking heterogeneity between regions and countries. National cardiology societies should advocate to their governments for urgent investment in standardised CR services.

## Introduction

Although the age-specific incidence of coronary heart disease (CHD) has declined in many regions of the world (1), demographic changes increase the societal burden of CHD and costs. In patients with established CHD the risk of recurrent events remains high; therefore secondary prevention strategies are essential and it has been shown that multi-component cardiac rehabilitation (CR) programmes results in better outcomes (2,3,4). Unfortunately CR is not offered to the majority of patients who suffer from a clinical CHD event. This has been clearly demonstrated in various European countries in the EUROASPIRE surveys (5,6) and also more recently in Norway (7) where only 14% of all patients who had been discharged with an acute myocardial infarction (AMI) during the period 2018–2021 participated in CR. The authors found only slightly lower CR activity during and after the COVID-19 pandemic (13.7%) compared to the years before (14.6%). At an international level less information is available on the provision, attendance, and effects of CR programmes. Some international CR registries have reported the provision of services using different methodologies and content but all depend on self reports with no direct contact with patients using, or not, such services (8).

INTERASPIRE is an international study of secondary prevention of coronary heart disease (CHD), undertaken in partnership with the World Heart Federation in 14 member countries drawn from all six WHO regions: Africa (Kenya, Nigeria and Tanzania), Americas (Argentina and Colombia), Eastern Mediterranean Region (Egypt and UAE), Europe (Poland and Portugal), South-East Asia (Indonesia), and Western Pacific Region (China, Malaysia, Philippines, Singapore) (9,10). The study was conducted in 2020–2023 through national societies of cardiology. The objective was to determine if international guideline targets for secondary prevention of CVD are being achieved and CR services provided together with their outcomes. The specific objectives of this study of CR were to determine:

- To what extent were patients referred to a CR facility following hospital discharge and which patient-specific factors are associated with referral?
- To what extent did such patients participate in a CR programme, if advised to do so, and which patient-related factors are associated with participation?
- Is participation in a CR programme associated with achieving lifestyle target and risk- factor control, cardioprotective drug use, and adherence?

## Material and methods

A detailed description of the study protocol for INTERASPIRE has been published (9,10). Within each country several geographical areas with a population greater than 500,000 people were selected and all public hospitals serving these populations were identified. A convenience sample of one or more hospitals from each region was taken. In brief, 4,548 consecutive patients, 18–80 years of age, with a first or recurrent coronary hospitalisation (elective or emergency coronary artery bypass surgery (CABG), elective or emergency percutaneous coronary intervention (PCI), AMI, and unstable angina/acute myocardial ischaemia) were identified. They were invited to a study visit at one of the 88 participating hospitals across 14 countries from the six WHO regions between six months and two years after the index hospitalisation (except for Africa where the timeframe was extended to three years to allow for lower incidence of the disease).

### Data collection

Data were collected by centrally trained research staff using standardised methods and instruments. Information on case history, demographics, health behaviours, CVD risk factor management and all prescribed medication was obtained from their medical records and from the patient interviews. Information regarding income level and occupational status was obtained from single questions during the interview. These data together with measurements and definitions of smoking habits, height and weight, waist circumference, blood pressure, and venous fasting blood analyses in a central laboratory have been published elsewhere (10).

Data were collected electronically and submitted online to the data management centre in the EURObservational Research Programme, European Heart House, Sophia Antipolis, France, for Argentina and Malaysia, and for the other countries, in the ARO Specialist Data Centre Services (previously AIMES), Liverpool, UK. Data were checked for completeness, internal consistency, and accuracy. All data were stored under the provisions of the national data protection regulations.

### Ethical considerations

Ethics approvals were obtained in all hospital centres and all patients provided written informed consent by means of a signed declaration.

### Statistical analyses

The frequencies of patient characteristics are presented as proportions and compared according to Fisher’s exact test. Multivariate analyses were done using logistic regression models. In contrast to the principal results paper the present analyses were conducted without direct standardization by age and sex. An alpha threshold of <0.05 was used to indicate statistical significance. All analyses were performed using SAS statistical software release V.9.4 (SAS Institute, Cary, NC) at the Department of Public Health and Primary Care, Ghent University, Ghent, Belgium.

## Results

A total of 4,548 patients participated in the survey (21% women), varying between countries from 126 in Kenya to 408 in Portugal. They were examined a year (median 1.05 year) after the index event. In Table 1, numbers of participating hospitals, numbers of recruited patients, and numbers and proportions of referrals to CR programmes are presented (overall and by country). In total, 34.4% of all patients reported having been advised to participate in a CR programme, with such reports varying by country from 4.0% in Kenya to 69.6% in Poland.

**Table 1:** Numbers of participating hospitals, numbers of recruited patients and numbers and proportions of patient-reported referral to a CR programme, by country.


	NUMBER OF CENTRES	NUMBER OF RECRUITED PATIENTS	REFERRAL TO A CR PROGRAMME*

Argentina	7	193	14.6% (28/192)

China	10	400	12.4% (49/396)

Colombia	7	399	56.2% (221/393)

Egypt	4	289	12.4% (34/274)

Indonesia	7	406	58.2% (235/404)

Kenya	3	126	4.0% (5/125)

Malaysia	5	402	28.4% (111/391)

Nigeria	12	242	29.4% (65/221)

Philippines	6	401	29.7% (116/390)

Poland	9	299	69.6% (208/299)

Portugal	6	408	39.4% (160/406)

Singapore	6	400	44.8% (174/388)

Tanzania	2	179	7.5% (13/174)

UAE	4	404	24.9% (65/261)

Total	88	4548	34.4% (1484/4314*)


*N = 234 patients answered ‘Don’t know’ to the question.

In Table 2 the associations between patient characteristics and referral to CR programmes in the 1,484 patients who reported being referred are described. There were no differences in CR referral rate by sex. Older patients aged ≥65 years were less frequently referred. Patients with elective PCI as the recruiting event were considerably less likely to be referred by comparison with patients following elective CABG. Patients with a lower educational level and those who were unemployed were also less likely to be referred.

**Table 2:** Association between patient characteristics and referral to a CR programme.


	REFERRED TO PARTICIPATE IN A CR PROGRAMME

**Sex**	

Male	35.0% (1193/3412)

Female	32.3% (291/902)

	*p = 0.13*

**Age at recruiting event**	

<55 years	35.7% (528/1480)

55–64 years	36.0% (567/1575)

≥65 years	30.9% (389/1259)

	*p = 0.0078*

**Recruiting event**	

Elective CABG	70.4% (209/297)

Elective PCI	25.5% (240/941)

Acute myocardial infarction STEMI	38.0% (528/1389)

Acute myocardial infarction Non-STEMI	38.0% (339/893)

Unstable angina/acute myocardial ischaemia	21.2% (168/794)

	*p < 0.0001*

Educational level*	

Low	23.9% (263/1102)

Intermediate	38.6% (832/2157)

High	37.2% (386/1039)

	*p < 0.0001*

**Occupational status**	

Full time employed	38.9% (742/1909)

Part time employed	36.5% (62/170)

Self employed	30.8% (224/728)

Unemployed	21.7% (48/221)

Retired	32.8% (334/1017)

Other	27.3% (72/264)

	*p < 0.0001*

**Hospitalizations prior to the recruiting event**	

Elective CABG	

No	34.2% (1429/4184)

Yes	42.0% (47/112)

	*p = 0.088*

Elective PCI	

No	34.4% (1249/3629)

Yes	33.5% (220/656)

	*p = 0.69*

STEMI or NSTEMI	

No	33.6% (1232/3669)

Yes	40.3% (213/528)

	*p = 0.0028*

Unstable angina/Acute myocardial ischaemia	

No	34.3% (1380/4024)

Yes	34.2% (68/199)

	*p = 0.99*

Angina pectoris	

No	34.5% (1360/3940)

Yes	32.3% (96/297)

	*p = 0.49*

Stroke	

No	34.4% (1438/4185)

Yes	37.5% (42/112)

	*p = 0.48*

Transient ischaemic attack	

No	34.2% (1460/4263)

Yes	38.5% (10/26)

	*p = 0.68*

Heart failure	

No	34.3% (1438/4197)

Yes	37.5% (33/88)

	*p = 0.57*

Peripheral artery disease	

No	34.4% (1460/4249)

Yes	30.3% (10/33)

	*p = 0.72*


*Note*: significances obtained by using Fishers’ exact test. *Educational level was categorised as low (primary school or lower), high (university or higher), or intermediate (all points between low and high).

Patients with a previous history of an AMI were more likely to be referred to a CR programme, but a personal history of other CV events, such as stroke or peripheral artery disease (PAD), was not associated with referral.

From multivariate analysis including age, recruiting events, educational level, and a personal history of AMI, referral rates to CR programmes were significantly lower in older patients, in those with elective PCI or unstable angina as the recruiting event, and in patients with a low educational level.

In Table 3 participation in a CR programme is presented by country among patients who reported being referred to participate in CR. Overall 57% of those referred participated in ≥50% of all sessions, 15% in less than 50%, and 27% did not participate at all. These proportions varied greatly between countries. Only 19.6% (847/4314) of all patients recruited to the study attended ≥50% of sessions (234 patients answered ‘Don’t know’ to the question were they referred to CR).

**Table 3:** Attendance in a CR programme by country.


	N	PATIENT ATTENDANCE*		

		NO	<50% OF SESSIONS	≥50% OF SESSIONS

Argentina	28	92.9% (26)	3.6% (1)	3.6% (1)

China	49	24.5% (12)	57.1% (28)	18.4% (9)

Colombia	221	17.6% (39)	14.9% (33)	67.4% (149)

Egypt	34	44.1% (15)	41.2% (14)	14.7% (5)

Indonesia	235	21.3% (50)	20.4% (48)	58.3% (137)

Kenya	5	0.0% (0)	40.0% (2)	60.0% (3)

Malaysia	111	30.6% (34)	9.9% (11)	59.5% (66)

Nigeria	64	70.3% (45)	9.4% (6)	20.3% (13)

Philippines	116	38.8% (45)	21.6% (25)	39.7% (46)

Poland	208	11.5% (24)	2.4% (5)	86.1% (179)

Portugal	160	15.0% (24)	6.3% (10)	78.8% (126)

Singapore	174	36.2% (63)	17.2% (30)	46.6% (81)

Tanzania	13	46.2% (6)	30.8% (4)	23.1% (3)

UAE	65	36.9% (24)	18.5% (12)	44.6% (29)

**All**	1483**	27.4% (407)	15.4% (229)	57.1% (847)


*Among those who were referred to a CR programme; **For 1 referred patient the information on the actual attendance in a CR programme was missing.

In Table 4 the patient characteristics associated with attendance of at least half of the sessions of a CR programme are presented. Good attendance did not differ by sex but was higher in older patients, those with elective CABG as the recruiting event, better educated patients, those retired or employed, and those with a higher income level.

**Table 4:** Association between patient characteristics and attendance in a CR programme (at least half of the sessions).


	ATTENDANCE IN A CR PROGRAMME % ATTENDING AT ≥50% OF THE SESSIONS

**Sex**	

Male	57.0% (680/1192)

Female	57.4% (167/291)

	*p = 0.95*

**Age at recruiting event**	

<55 years	53.5% (282/527)

55–64 years	57.0% (323/567)

≥65 years	62.2% (242/389)

	*p = 0.031*

**Recruiting event**	

Elective CABG	68.9% (144/209)

Elective PCI	52.5% (126/240)

AMI STEMI	56.6% (299/528)

AMI Non-STEMI	61.8% (209/338)

Unstable angina/myocardial ischaemia	41.1% (69/168)

	*p < 0.0001*

**Educational level**	

Low	45.6% (120/263)

Intermediate	59.9% (498/832)

High	58.8% (227/386)

	*p = 0.00021*

**Occupational status**	

Full time employed	59.1% (438/741)

Part time employed	53.2% (33/62)

Self employed	41.1% (92/224)

Unemployed	41.7% (20/48)

Retired	67.7% (226/334)

Other	52.8% (38/72)

	*p < 0.0001*

**Income**	

Low	45.5% (171/376)

Middle	59.0% (566/960)

High	66.7% (54/81)

	*p < 0.0001*


The content of the CR programmes as reported by patients is presented in Table 5. A supervised exercise programme was the most frequently reported component, but the heterogeneity in reported content is the most striking observation between countries.

**Table 5:** Content of the cardiac rehabilitation programme (reported by the patients).


	*PLEASE SPECIFY WHAT YOU RECEIVED AS PART OF THIS PROGRAMME*						

	WRITTEN MATERIALS	SUPERVISED EXERCISE PROGRAMME	HEALTH PROMOTION WORKSHOPS	STRESS MODIFICATION AND RELAXATION	DIET/WEIGHT MODIFICATION	SMOKING CESSATION*	OTHER

Argentina	50.0% (1)	50.0% (1)	100.0% (2)	0.0% (0)	0.0% (0)	—	50.0% (1)

China	10.8% (4)	75.7% (28)	5.4% (2)	8.1% (3)	24.3% (9)	50.0% (4)	0.0% (0)

Colombia	46.7% (85)	50.5% (92)	36.3% (66)	17.6% (32)	29.7% (54)	9.7% (3)	0.0% (0)

Egypt	68.4% (13)	47.4% (9)	42.1% (8)	52.6% (10)	63.2% (12)	45.5% (5)	0.0% (0)

Indonesia	22.2% (41)	94.1% (174)	44.9% (83)	33.5% (62)	47.0% (87)	36.8% (28)	0.5% (1)

Kenya	20.0% (1)	80.0% (4)	20.0% (1)	20.0% (1)	60.0% (3)	—	0.0% (0)

Malaysia	46.8% (36)	79.2% (61)	55.8% (43)	40.3% (31)	46.8% (36)	52.2% (12)	0.0% (0)

Nigeria	31.6% (6)	10.5% (2)	47.4% (9)	15.8% (3)	42.1% (8)	50.0% (1)	5.3% (1)

Philippines	47.8% (33)	65.2% (45)	33.3% (23)	26.1% (18)	40.6% (28)	17.4% (4)	1.4% (1)

Poland	72.8% (134)	91.3% (168)	59.2% (109)	76.6% (141)	79.3% (146)	52.5% (32)	1.1% (2)

Portugal	64.7% (86)	91.7% (122)	60.9% (81)	60.2% (80)	72.2% (96)	59.7% (37)	1.5% (2)

Singapore	70.3% (78)	83.8% (93)	33.3% (37)	11.7% (13)	26.1% (29)	21.7% (5)	1.8% (2)

Tanzania	28.6% (2)	28.6% (2)	28.6% (2)	28.6% (2)	57.1% (4)	—	0.0% (0)

UAE	63.4% (26)	41.5% (17)	29.3% (12)	19.5% (8)	24.4% (10)	16.7% (2)	0.0% (0)

Total	51.0% (546)	76.4% (818)	44.6% (478)	37.7% (404)	48.7% (522)	40.1% (133)	0.9% (10)


*For patients smoking in the month prior to hospital admission.

The prevalence of lifestyle risk factors according to attendance at CR programmes are shown in Table 6. Smoking, persistent smoking, and physical inactivity were less frequent in patients who participated in ≥50% of all sessions as compared to those who did not, or attended <50% of sessions.

**Table 6:** Lifestyle related characteristics according to attendance at CR programmes.


	ATTENDANCE (IF REFERRED)		

	NO OR <50% OF SESSIONS	≥50% OF SESSIONS	SIGNIFICANCE

Smoking^1^, %	20.8% (132/636)	13.2% (112/847)	p = 0.0001

Persistent smoking^2^, %	54.0% (122/226)	40.3% (104/258)	p = 0.0034

Overweight^3^, %	64.2% (408/636)	66.9% (566/846)	p = 0.27

Obesity^4^, %	21.9% (139/636)	22.9% (194/846)	p = 0.66

Central overweight^5^, %	65.4% (415/635)	68.0% (575/845)	p = 0.29

Central obesity^6^, %	33.4% (212/635)	34.6% (292/845)	p = 0.66

Physical inactivity^7^, %	64.5% (410/636)	52.4% (444/847)	p < 0.0001


^1^Self-reported current smoking or >10 ppm carbon monoxide in breath; ^2^Self-reported smoking or >10 ppm carbon monoxide in breath in patients reporting to be a smoker in the month prior to the recruiting event; ^3^Body mass index (BMI) ≥25 kg/m^2^; ^4^BMI ≥30 kg/m^2^; ^5^ Waist circumference ≥80 cm for women and ≥94 cm for men (South Asian and Chinese men ≥90 cm); ^6^Waist circumference ≥88 cm for females and ≥102 cm for males; ^7^Not performing regular physical activity ≥30 minutes on average 5 times a week

In Table 7 (a) the patients reporting attendance at CR programmes if referred compares non-participants (none or <50% of sessions) and participants (≥50% of sessions) regarding blood pressure levels, LDL-cholesterol levels and variables related to diabetes. In Table 7 (b) only those on prescribed drug treatment for hypertension, dyslipidemia or diabetes in the two groups are compared. For all patients, as well as for those on drug therapy blood pressure and LDL-cholesterol was better controlled among those attending ≥50% of the sessions, but a majority of patients did not achieve any of the guideline risk factor targets.

**Table 7:** Achievement of blood pressure, LDL-cholesterol and HbA1c targets.


	ATTENDANCE (IF REFERRED)		

	NO OR <50% OF SESSIONS	≥50% OF SESSIONS	SIGNIFICANCE

**(a) All patients**			

Systolic/diastolic BP			

≥130/80 mmHg, %	62.9% (400/636)	53.8% (456/847)	p = 0.0006

≥140/90 mmHg, %	33.3% (212/636)	25.6% (217/847)	p = 0.0014

≥160/100 mmHg, %	8.5% (54/636)	5.8% (49/847)	p = 0.049

LDL cholesterol			

≥1.4 mmol/L, %	86.7% (510/588)	79.9% (617/772)	p = 0.0011

≥1.8 mmol/L, %	65.3% (384/588)	57.1% (441/772)	p = 0.0025

≥2.5 mmol/L, %	32.3% (190/588)	27.2% (210/772)	p = 0.041

Diabetes^1^			

Self-reported, %	43.7% (278/636)	39.0% (330/847)	p = 0.070

Newly diagnosed diabetes^2^, %	12.0% (36/299)	8.3% (29/348)	p = 0.15

IGT^3^, %	19.7% (59/299)	26.0% (107/412)	p = 0.059

**(b) Treated patients**			

Systolic/diastolic blood pressure^4^			

<130/80 mmHg, %	38.1% (219/575)	45.8% (366/799)	p = 0.0048

<140/90 mmHg, %	68.0% (391/575)	74.1% (592/799)	p = 0.015

<160/100 mmHg, %	91.8% (528/575)	93.9% (750/799)	p = 0.16

LDL cholesterol^5^			

<1.4 mmol/L, %	14.1% (72/510)	20.9% (150/716)	p = 0.0026

<1.8 mmol/L, %	36.9% (188/510)	44.6% (319/716)	p = 0.0081

<2.5 mmol/L, %	70.2% (358/510)	74.9% (536/716)	p = 0.078

HbA1c in patients with diabetes^6^			

<53 mmol/mol (7.0%), %	54.9% (135/246)	62.9% (188/299)	p = 0.066

<64 mmol/mol (8.0%), %	78.0% (192/246)	82.9% (248/299)	p = 0.16


^1^Including those reporting the use of glucose lowering drugs; ^2^According to OGTT-data: fasting glucose ≥7.0 mmol/L and/or 2h-postload glucose ≥11.1 mmol/L in patients without self-reported diabetes; ^3^IGT impaired glucose tolerance: Fasting plasma glucose <7.0 mmol/l and 2h glucose ≥7.8 and <11.1 mmol/l in patients without self-reported diabetes; ^4^In patients using beta-blockers, ACE inhibitors, angiotensin II receptor blockers, renin inhibitors, calcium channel blockers, diuretics or other anti-hypertensive drugs; ^5^In patients using statins, fibrates, nicotinic acid, cholesterol absorption inhibitors, PCSK9 inhibitors, resins or a fixed-dose combination of lipid lowering drugs; ^6^In patients with self-reported diabetes or using insulin, metformin, sulphonylurea, incretins (gliptins and/or GLP1 analogs), glinides, glitazones, SGLT2 inhibitors or alpha-glucosidase inhibitors.

In Table 8 non-participants are compared with participants in the use of cardioprotective drugs and medication adherence. Participants report higher prescription rates for beta-blockers, ACE inhibitors/ARBs, lipid-lowering drugs and the ‘four drug pillars of secondary prevention’ defined as follows: (1) antiplatelets or anticoagulants including NOACs, (2) beta-blockers, (3) angiotensin-converting enzyme inhibitors (ACE inhibitors) or angiotensin II receptor blockers (ARBs), (4) lipid-lowering drugs. Medication adherence is also significantly higher for lipid-, blood-pressure-, and glucose-lowering drugs in particpants compared to non-participants.

**Table 8:** Reported cardio-protective medication and medication adherence comparing non-participants with participants.


	ATTENDANCE (IF REFERRED)		

	NO OR <50% OF SESSIONS	≥50% OF SESSIONS	SIGNIFICANCE

Antiplatelets/anticoagulants^1^, %	94.9% (600/632)	95.2% (805/846)	p = 0.90

Beta-blockers, %	78.0% (492/631)	83.7% (707/845)	p = 0.0058

ACE inhibitors/ARBs^2^, %	65.3% (413/632)	70.6% (597/846)	p = 0.036

Lipid-lowering drugs^3^, %	87.8% (555/632)	92.9% (786/846)	p = 0.0010

The four drug pillars’, %	49.8% (314/631)	56.9% (481/845)	p = 0.0071

High-intensity statins, %	61.9% (390/630)	64.4% (542/842)	p = 0.35

Medication adherence*			

Lipid-lowering drugs, %	90.8% (545/600)	96.1% (788/820)	p < 0.0001

Blood-pressure-lowering drugs, %	91.9% (514/559)	96.9% (757/781)	p < 0.0001

Glucose-lowering drugs, %	91.6% (274/299)	95.8% (367/383)	p = 0.034


^1^Including NOACs; ^2^Including renin inhibitors; ^3^Statins, fibrates, nicotinic acid, cholesterol absorption inhibitors, PCSK9 inhibitors, resins, or a fixed-dose combination of lipid-lowering drugs.

The prevalence of an abnormal HADS depression score ≥11 was significantly higher in the non-participants (8.8%) compared with the participants (4.0%) (p = 0.0002). For the abnormal HADS anxiety score ≥11 the results were: 10.8% in non-participants vs 8.0% in participants (p = 0.07).

In Table 9 non-participants are compared with participants for the INTERASPIRE Guideline Target Score, a published 10-point summary score based on lifestyle, risk factors, and medications (10). The distribution of the score is significantly better in participants compared with non-participants.

**Table 9:** Distribution of the INTERASPIRE Guideline Target Score for achieving lifestyle, risk factor, and therapeutic targets.


	PARTICIPATION (IF ADVISED)		

	NO OR <50% OF SESSIONS	≥50% OF SESSIONS	SIGNIFICANCE

Guideline Target Score			p < 0.0001

0–5, %	26.5% (152/574)	14.4% (109/757)	

6–7, %	49.0% (281/574)	50.1% (379/757)	

8–10, %	24.6% (141/574)	35.5% (269/757)	


The Guideline Target Score is from 0 to 10 and is defined as follows. For each of the 10 items listed below either 1 or 0 is awarded. Lifestyle: Non-smoker or ex-smoker validated with breath CO < 10 ppm; BMI < 25 kg/m^2^; Performing regular physical activity ≥30 minutes on average 5 times a week; Risk factors: BP < 140/90 mmHg; LDL-C < 1.8 mmol/L; HbA1c < 6.5% (48 mmol/mol) and FG < 5.5 mmol/l (and in patients with diabetes HbA1c < 7% (53 mmol/mol) and FG < 7 mmol/l); Therapeutics: antiplatelet therapies or anticoagulants; beta blockers; RAAS inhibitors; lipid-lowering drugs.

## Discussion

This international study of cardiac rehabilitation (CR) across all six WHO regions shows that despite a Class 1 recommendation in all cardiovascular prevention guidelines the majority of patients with CHD were not referred and did not attend CR. Most strikingly there is enormous heterogeneity between countries with an up to 16-fold difference (4.0% to 69.6%) in referral between countries. The reasons for this enormous variation in service provision are complex and likely to be very different both between countries, and between regions and hospitals within countries, but the startling fact is that in every country not all patients are referred to CR, and in most countries it is only a minority.

CR is a multicomponent model of care including exercise training and physical activity promotion, health education, cardiovascular risk factor management, and psycho-social support, personalised to the individual needs of patients diagnosed with heart disease (11). A wealth of scientific evidence supports CR as a clinically and cost-effective intervention for patients with CHD. CR results in better control of CVD risk factors and improved quality of life as demonstrated in randomised controlled trials as well as meta-analyses of observational cohort studies (4,11,12,13,14,15,16). An updated Cochrane Review on exercised-based CR supports the conclusion that CR provides important benefits to people with CHD, including reduced risk of MI, a likely small reduction in all-cause mortality, and a large reduction in all-cause re-hospitalisation, along with associated healthcare costs, and improved health-related quality of life up to 12 months’ follow-up (4).

One of the major challenges facing CR is the heterogeneity in service provision for patients with CHD (17). In the INTERASPIRE study only a third of the patients reported at the interview having been referred to CR programmes. In a follow-up investigation of CR services, 12 out of the 88 participating hospitals (representing 496 patients or 17.5% of all those who did not report referral to CR programmes) reported they did not offer any CR programmes or refer patients to facilities offering CR. The impact of this observation on the final proportion being able to attend, but not doing so, increases the referral rate from 34.4% to 38.5% where CR services were available which is still a minority of patients. The low referral rates may have been influenced by the COVID-19 pandemic, but we were not able to establish with any certainty with the lead cardiologists at each hospital whether this was the case or not. Other factors will have included the attitude of cardiologists and other health care professionals about the value of CR and patient perceptions of such a service as well.

All patients should be eligible for CR and referral bias should not exist. However, older patients, those with a lower educational level and unemployed status were not referred. In addition, the event itself was associated with referral bias with patients following CABG being more likely to be referred. About a third of all patients were referred to a programme, but only 57.1% of these attended more than half of the sessions and almost a quarter did not attend at all. Attendance rates were higher in older patients, those with elective CABG as the recruiting event, those with a higher educational level, those retired or employed and in those with a self reported higher income level which will be a factor if CR services are charged for outside the public sector. In the principal results paper of INTERASPIRE we reported 9% attendance at CR across all 14 countries combined, but this was an age- and sex-standardized proportion, which explains this apparent difference with the present results.

The evolution of CR into a comprehensive programme encompassing both secondary prevention and rehabilitation is not reflected in the content of the CR programmes as reported by the patients. The top component was supervised exercise programmes (reported by three quarters of patients), followed by the provision of written educational material, diet/weight modification programmes, health promotion workshops, smoking cessation sessions for those who reported smoking at the time of the event, and finally stress modification and relaxation sessions (reported by only a third). The most striking observation is the enormous heterogeneity in reported programme content, even for supervised exercise sessions, between countries. In our systematic review and meta-analysis of contemporary randomized controlled trials of prevention and rehabilitation we found that comprehensive programmes (managing six or more risk factors) and those prescribing and monitoring medications within programmes to lower blood pressure and lipids both reduced cardiovascular events and total mortality (18).

Therefore, although there were some improvements in the lifestyle of those fully participating in CR the prevalence of persistent smoking still remained high at 40%. There was no impact on obesity and central obesity and, although physical inactivity was reduced, over half of these patients were still classified as inactive. The HADS depression score was also significantly lower in those participating in CR. Although there was no mention of managing risk factors—blood pressure, lipids or glucose—to defined targets, or prescribing and maximising adherence with cardioprotective medications in the patients description of programmes there were significant improvements in the control of blood pressure and LDL-C among patients attending more than half the CR sessions. The four drug pillars for secondary prevention of CVD—anti-platelets, beta-blockers, ACE inhibitors/ARBs and statins—were prescribed more frequently in those attending more sessions, and specifically this applied to beta-blockers, ACE inhibitors/ARBs and statins, but not high-intensity statins. Yet only a minority of patients had achieved the LDL-C target of <1.4 mmol/l. Medication adherence was also reported to be higher for blood pressure, lipid, and diabetes management in those attending more sessions. The INTERASPIRE Guideline Target Score, a 10-point score summarising achievement of lifestyle, risk factor and therapeutic targets, was also higher in those attending more sessions.

The results of INTERASPIRE are in accordance with other observational studies showing that access to CR remains poor with between 5–50% of all eligible patients with coronary disease receiving rehabilitation despite Class 1 guideline recommendations (11,19,20,21,22,23). Global availability and inequity in CR provision was quantified by the International Council of Cardiovascular Prevention and Rehabilitation (ICCPR) audit (8,23,24). The ICCPR study revealed that CR is available in only half of the countries of the world, and this geographical distribution of CR is negatively correlated with the incidence of ischaemic heart disease, according to the Global Burden of Disease study (25). The densities in CR provision were estimated to be only one CR place available for every 66 patients with CHD in low- and middle-income countries, compared with one place for every 3.4 patients in high-income countries (23,24). CR practice faces major challenges globally in service provision and access, and as a consequence all too many patients have a poorer quality of life and a shorter life expectancy.

## Strengths and limitations

The strength of INTERASPIRE is that it uses standardised methodology to characterise patients with CHD and how they were managed. This investigation is comprehensive because it includes aspects of lifestyle together with anxiety and depression, risk factor management and prescriptions of cardioprotective medications. Even if the low referral rate to CR may partly be due to the COVID-19 pandemic, the results are consistent with the ICCPR report showing wide variation and low provision of CR globally. The selected regions and hospitals in each country may not be nationally representative and may result in selection bias. However any bias is likely to be conservative as less specialised hospitals are less likely to offer CR and sicker patients may be less willing to attend CR, and therefore, a truly representative picture of patient management is likely to be even poorer. The use of self-reported data and the measures to assess physical activity may be a limitation but the interviews were all conducted by centrally trained research workers using the same questionnaire and some of the lifestyle factors are also reflected in more objective measures using standardised instruments (e.g breath CO) by the interviewers.

## In conclusion

INTERASPIRE provides a unique picture of CR provision using standardised methodology in patients with CHD. It demonstrates that referral to and attendance at CR varies enormously between countries despite CR being a Class 1 recommendation in all international guidelines. Only one third of patients reported being advised to attend any form of CR with just one in five patients attending 50% of the sessions. This resulted in poor patient outcomes in terms of lifestyle, risk factor control, and drug prescriptions. To improve patient outcomes national cardiology societies and patients associations should advocate for urgent investment in CR services.

## Data Accessibility Statement

The data underlying this article will be shared on reasonable request to the corresponding author. The scientific protocol is publicly available through the website of the National Institute for Prevention and Cardiovascular Health http://www.nipc.ie. Aggregated patient data from INTERASPIRE will be made available after publication of principal results through the World Heart Observatory (world-heart-federation.org/world-heart-observatory/) of the World Heart Federation. Individual patient data will be returned to the participating national societies of cardiology for advocacy at a national level.

## Additional File

The additional file for this article can be found as follows:

10.5334/gh.1458.s1 Supplementary File. Supplementary Table 1 and Appendix: Study centers and collaborators.
