# Supplementary material for: Cardiac Rehabilitation in Patients with Coronary Heart Disease – Provision, Attendance, and Outcomes: Results from the INTERASPIRE Survey from Fourteen Countries Across Six WHO Regions
Source: Glob Heart. 2025 Sep 2;20(1):75. doi: 10.5334/gh.1458 (PMC12412450; doi:10.5334/gh.1458)
Supplement: Supplementary File. — Supplementary Table 1 and Appendix: Study centers and collaborators. [file gh-20-1-1458-s1.pdf]

Supplementary table 1. Lifestyle related characteristics, risk factors and medication use according to attendance at CR programmes

	Attendance (if referred)		Odds Ratio (95% CI), P-value	
	No or < 50% of sessions	≥ 50% of sessions	Crude	Adjusted for age and sex
Smoking, %	20.8% (132/636)	13.2% (112/847)	0.58 (0.44-0.77), P=0.00012	0.61 (0.46-0.81), P=0.00063
Persistent smoking, %	54.0% (122/226)	40.3% (104/258)	0.58 (0.40-0.83), P=0.0027	0.57 (0.40-0.82), P=0.0027
Overweight, %	64.2% (408/636)	66.9% (566/846)	1.13 (0.91-1.40), P=0.269	1.16 (0.93-1.44), P=0.186
Obesity, %	21.9% (139/636)	22.9% (194/846)	1.06 (0.83-1.36), P=0.623	1.10 (0.86-1.41), P=0.447
Central overweight, %	65.4% (415/635)	68.0% (575/845)	1.13 (0.91-1.40), P=0.276	1.12 (0.90-1.40), P=0.309
Central obesity, %	33.4% (212/635)	34.6% (292/845)	1.05 (0.85-1.31), P=0.638	1.06 (0.84-1.32), P=0.638
Physical inactivity, %	64.5% (410/636)	52.4% (444/847)	0.61 (0.49-0.75), P<0.0001	0.59 (0.47-0.73), P<0.0001
Systolic/diastolic BP ≥ 140/90 mmHg, %	33.3% (212/636)	25.6% (217/847)	0.69 (0.55-0.86), P=0.0012	0.66 (0.53-0.83), P=0.00037
LDL cholesterol ≥ 1.8 mmol/L, %	65.3% (384/588)	57.1% (441/772)	0.71 (0.57-0.88), P=0.0022	0.71 (0.57-0.88), P=0.0023
Self-reported diabetes, %	43.7% (278/636)	39.0% (330/847)	0.82 (0.67-1.01), P=0.066	0.79 (0.64-0.98), P=0.033
HbA1c <7.0% in patients with diabetes	54.9% (135/246)	62.9% (188/299)	1.39 (0.99-1.96), P=0.059	1.45 (1.03-2.06), P=0.036
Antiplatelets/anticoagulants, %	94.9% (600/632)	95.2% (805/846)	1.05 (0.65-1.68), P=0.849	1.08 (0.67-1.73), P=0.766
Beta-blockers, %	78.0% (492/631)	83.7% (707/845)	1.45 (1.11-1.88), P=0.0057	1.45 (1.12-1.89), P=0.0054
ACE inhibitors/ARBs, %	65.3% (413/632)	70.6% (597/846)	1.27 (1.02-1.59), P=0.033	1.25 (1.00-1.56), P=0.047
Lipid-lowering drugs, %	87.8% (555/632)	92.9% (786/846)	1.82 (1.27-2.59), P=0.00096	1.81 (1.27-2.59), P=0.0011
'The four drug pillars' , %	49.8% (314/631)	56.9% (481/845)	1.33 (1.08-1.64), P=0.0064	1.33 (1.08-1.63), P=0.0076
High intensity statins, %	61.9% (390/630)	64.4% (542/842)	1.11 (0.90-1.38), P=0.331	1.12 (0.90-1.38), P=0.318
Adherence to lipid lowering drugs, %	90.8% (545/600)	96.1% (788/820)	2.49 (1.59-3.89), P<0.0001	2.48 (1.58-3.90), P<0.0001
Adherence to BP lowering drugs,%	91.9% (514/559)	96.9% (757/781)	2.76 (1.66-4.59), P<0.0001	2.80 (1.68-4.65), P<0.0001
Adherence to glucose lowering drugs, %	91.6% (274/299)	95.8% (367/383)	2.09 (1.10-4.00), P=0.025	2.12 (1.11-4.05), P=0.023
Guideline Target Score 8-10, %	24.6% (141/574)	35.5% (269/757)	1.69 (1.33-2.16), P<0.0001	1.72 (1.35-2.20), P<0.0001

Appendix

INTERASPIRE was undertaken in partnership with the Asian Pacific Society of Cardiology, European Atherosclerosis Society, European Society of Cardiology, InterAmerican Society of Cardiology, International Atherosclerosis Society, PanAfrican Society of Cardiology and World Heart Federation. The structure of the administrative organisation is described below followed by a list of participating study centres and organisations, principal investigators and other research personnel.

Scientific Steering Committee:
Executive Committee: JW McEvoy (Galway, Ireland, Co-chair INTERASPIRE Executive Committee) D Wood (Galway, Ireland, Co-chair INTERASPIRE Executive Committee) A Adamska (Galway, Ireland), G De Backer (Ghent, Belgium), D De Bacquer (Ghent, Belgium), I Erlund (Helsinki, Finland), S Ganly (Galway, Ireland), C Jennings (Galway, Ireland), K Kotseva (Galway, Ireland), G.Y.H Lip (Liverpool, UK), K Ray (London, UK), L Rydén (Stockholm, Sweden)

National Co-ordinators: A Abreu (Lisbon, Portugal), W Almahmeed (Abu Dhabi, UAE), AM Ambari (Jakarta, Indonesia), JB Ge (Shanghai, China), H Hasan-Ali (Assiut, Egypt), Y Huo (Beijing, China), P Jankowski (Warsaw, Poland), R Jimenez (Manila, Philippines), Y Li (Shanghai, China), AS Mahmood Zuhdi (Kuala Lumpur, Malaysia), A Makubi (Dar es Salaam, Tanzania), AC Mbakwem (Lagos, Nigeria), L Mbau (Nairobi, Kenya), JL Navarro Estrada (Buenos Aires, Argentina), OS Ogah (Ibadan, Nigeria), E Ogola (Nairobi, Kenya), A Quintero-Baiz (Barranquilla, Colombia), MU Sani (Kano, Nigeria), MI Sosa Liprandi (Buenos Aires, Argentina), JWC Tan (Singapore, Singapore), M Urina Triana (Barranquilla, Colombia), TJ Yeo (Singapore, Singapore)

Coordinating centre: National Institute for Prevention and Cardiovascular Health, National University of Ireland – Galway, Ireland: JW McEvoy, D A Wood, A Adamska, C Jennings, K Kotseva, S Ganly, A Zekic

Diabetes centre: Department of Cardiology, Karolinska University Hospital, Stockholm, Sweden: L Rydén

Data management centres: EURObservational Research Programme Department, European Heart House, Sophia Antipolis, Nice, France: D Salako, C Toulouse, G Chhabra; AIMES Management Services Limited, Liverpool, UK: R Spragg, M Sullivan

Statistical analysis centre: Department of Public Health and Primary Care, University of Ghent, Belgium: D De Bacquer

Central laboratory: Biomarkers, Department of Government Services, Finnish Institute for Health and Welfare, Helsinki, Finland: I Erlund, E Jarvensivu, T Vihervaara

Study centres, organisations, investigators and other research personnel (the National Co-ordinators in each country are indicated by asterisk, the PIs in each centre are indicated by double asterisk):

Americas

Argentina

Argentine Society of Cardiology, HA Deschle
JL Navarro Estrada*, MI Sosa Liprandi*
Sanatorio Mitre, Buenos Aire: S Bellia**
Sanatorio Güemes, Buenos Aires: R Villarreal**, M Elfman, J Perea,
Hospital Santojanni, Buenos Aires: F Lombardi**
Hospital Italiano, Buenos Aires: W Masson**, B Castaño, A Gamarra, M Llamedo
INCOR, La Rioja: P Santander**, A Barco, V Flores, C Leyes, F Rodríguez
Sanatorio Pasteur, San Fernando del Valle de Catamarca: MP Marturano**, WR Alustiza
Fundación Favaloro, Buenos Aires: E Duronto**, G Procopio
Hospital Alemán: C Higa**, O Corigliano, MA Sanchez

Colombia

Sociedad Colombia de Cardiologia y Cirugia Cardiovascular, F del Cristo Mendoza Beltrán, P Luna
MA Urina Triana*, A Quintero-Baiz *
Clinica Centro, Barranquilla: C Renowitsky, M Pulgar
Clinica Cardio Colombia, Bogota: J Lievano**, A Hernandez, G Cordoba
Clinica CES, Medellin: M Duque**, L Duque
Fundacion Clinica Shaio, Bogota: F Mendoza**, J Rojas
Clinica Las Americas, Medellin: N Jaramillo**, S Zuluaga, A Restrepo, J Munos
Fundacion Santa Fe, Bogota: A Buitrago**, H Ramirez, F Figueroa, C Gonzales
Clinica de La Costa, Barranquilla: A Cadena**, S Bermudez, L Gomez

South-East Asia

Indonesia

Indonesian Heart Association, I Firdaus
AM Ambari*
National Cardiovascular Center Harapan Kita, Department of Cardiology and Vascular Medicine Universitas Indonesia, Jakarta: B Dwiputra**, D Arityanti, E Susilowati
Dr. M. Djamil General Hospital, Department of Cardiology and Vascular Medicine Universitas Andalas, Padang: R Hamdani**, CK Krevani, M Syaoqi
Dr. Zainoel Abidin General Hospital, Department of Cardiology and Vascular Medicine Universitas Syiah Kuala, Banda Aceh: M Ridwan**, M Fitra ND, HA Sariningrum
Dr. Sardjito General Hospital, Department of Cardiology and Vascular Medicine Universitas Gadjah Mada, Yogyakarta: IA Arso**, AB Hartopo, VY Anggraeni
H. Adam Malik General Hospital, Department of Cardiology and Vascular Medicine Universitas Sumatera Utara, Medan: AH Raynaldo**, H Hasan, YF Siregar

Dr. Saiful Anwar General Hospital, Department of Cardiology and Vascular Medicine Universitas Brawijaya, Malang: CT Tjahjono**, FA Sihotang, G Erickatulistiawan
Dr. Hasan Sadikin General Hospital, Department of Cardiology and Vascular Medicine Universitas Padjadjaran, Bandung: BB Tiksnadi**, M Febrianora, NF Tarsidin

Malaysia
National Heart Association of Malaysia, AYY Fong, WA Wan Ahmad
AS Mahmood Zuhdi*
University Malaya Medical Centre, Kuala Lumpur: AS Mahmood Zuhdi**, WA Wan Ahmad, MF Hadi, Z Abu Kassim, NA Shaharudin, MN Mohamad Sithik, I Mohd Moussa
Sarawak Heart Centre, Kota Samarahan: AYY Fong**, TK Ong
Sarawak General Hospital, Kuching: DHP Foo**, AYY Fong, RHC Jong
Hospital Sultanah Aminah, Johor Bahru: CY Lee**, HS Kim, G Mahadevan
Hospital Pulau Pinang, George Town: MASK Abdul Kader**, N Abdul Aziz
Hospital Queen Elizabeth II, Kota Kinabalu: HB Liew**, EB Jam, D Regaibalan

Philippines
Philippine Heart Association, O Bugarin, A Leus, A Baniqued, J Arellano, LG Mendoza
R Jimenez*
St Lukes Medical Center, Taguig City: R Jimenez**, R Mendoza, M Reyes
St Lukes Medical Center, Quezon City: S Locnen**, D Luna, M Reyes
Philippine General Hospital, Manila: R Tiongco II**, P Alad, J Besa
St Paul's Hospital, Iloilo City: L Tirador**, E Estoce, A Sian, R Roque
Chong Hua Hospital, Cebu City: A Montejo**, A Discípulo, H Lastimosa
Davao Doctors Hospital, Davao City: E Ybanez**, A Bernan, R Coronel, MJ Samson III, T Tamayo

Singapore
Singapore Heart Foundation, HC Tan
JWC Tan*, TJ Yeo*
National University Hospital, Singapore: TJ Yeo**, M Dalakoti, NF Md Fadzillah, SL Lim, TT Low, JSY Ong, JHY Ting, LYT Wang, CY Wong
National Heart Centre Singapore, Singapore: JS Ho**, FY Chee, C Cheng, CH Koh, D Yip
Sengkang General Hospital, Singapore: ZJ Huang**, CY Chin, K Chua, JM Fam, M Idu, YS Keh, CY Lim, M Rizwan, JWC Tan, C Tsang, NY Wong, JJL Yap, MTS Tan
Changi General Hospital, Singapore: JJ Tan**, LH Goh, KH Tan, YM Wong, YR Yee, MA Yuslane
Khoo Teck Puat Hospital, Singapore: YS Weng**, CP Wong, SS Imran, ZY Lim, TM Lu, JL Su
Tan Tock Seng Hospital, Singapore: V Hoon**, YT Ho, SJL Quek

Eastern Mediterranean Region
Egypt

Egyptian Society of Cardiology, I Shawki
H Hasan-Ali*
Assiut University Heart Hospital, Assiut: H Hasan-Ali**, M Abdelmegid, A Marghany, E Mosad, EA Shafik
Aswan University Hospital, Aswan: A Ibrahim**, R Ghaleb, E Kholef, BE Shamandy
Zagazig University Hospital, Zagazig: T Moustafa**, M Sobeih, M Mortada
Banha University Hospital, Banha: M Elbarbary**, M Elawady

United Arab Emirates
Emirates Cardiac Society, Juwairia Al Ali
W Almahmeed*
Cleveland Clinic Abu Dhabi, Abu Dhabi: S Hashmani**, Y Manla, CM Green, R Garrod
Sheikh Shakhbout Medical City, Abu Dhabi: S Nour**, A Firas Alhammadi, Y Mohamed, R Isaac
Dubai Academic Health Corporation, Dubai: N Bazargani**, A Almulla, J K Praveen
Rashid Hospital, Dubai: JY Al Ali**, OA Osman Bashir, AK Faraj, SS Hashmath, R Lamine Hamadi, RE Ahmad, MS Prabakaran

Western Pacific Region
China
Chinese Cardiovascular Association, XR JI
JB Ge*, Y Huo*, Y Li*
Zhongshan Hospital Fudan University, Shanghai: JB Ge**, C Wang, G Zhao, SL Mi, YF Peng, ZY Qi
Fudan University Huashan Hospital, Shanghai: Y Li**, LW Bao, W Gao
Peking University First Hospital, Beijing: Y Huo**, B Zheng, J Fang,
The Second Hospital of Hebei Medical University, Shijiazhuang: XS Gu**, LF Su, Q Wang, S Tong, XY Chen, Y Fu
The First Affiliated Hospital of Harbin Medical University, Harbin: Y Li**, J Shi, JS Jing, L Sheng, MJ He, S Wang, W Pan, YH Kong
Tianjin Chest Hospital, Tianjin University, Tianjin: HL Cong**, YY Zhang
Affiliated Hospital of Zunyi Medical University, Zunyi: JL Chen**, B Shi, RZ Zhao, W Zhang
Dongguan Hospital of Guangzhou University of Chinese Medicine, Dongguan: JF Ye**, CJ Hou, HY Huang
The 920th Hospital of Joint Logistics Support Force of the Chinese People's Liberation Army, Kunming: LX Yang**, L Chang, ZH Yang
Fuwai Central China Cardiovascular Hospital, Zhengzhou: CY Gao**, JH Zhang, Y Zhang

Africa
Kenya
Kenya Cardiac Society, B Samia
National Coordinators: E Ogola*, L Mbau*

Coast General Teaching and Referral Hospital, Mombasa: K Mwazo**, A Bajaber, B Nduati, J Mkilo, M Mwasha, M Sood
Moi Teaching and Referral Hospital, Eldoret: F Barasa**, P Biwott
Kenyatta National Hospital, Nairobi: B Gitura**, P Kinuthia

Nigeria
Nigerian Cardiac Society, OS Ogah
OS Ogah*, AC Mbakwem*, MU Sani*
University College Hospital, Ibadan: OS Ogah**, OA Orimolade, EC Omoruyi, OZ Makinde, VO Ojo, F Otesanya
Lagos University Teaching Hospital, Lagos: AC Mbakwem**, CE Amadi, S Ogedegbe
University of Nigeria Teaching Hospital Ituku-Ozalla, Enugu: EC Ejim**, AB Odom, N Udora
Federal Medical Centre, Umuahia, Abia State: CJ Maduka**, J Onyemachi
University of Port Harcourt Teaching Hospital, Port Harcourt: EA Edafe**, ST Dodiyi-Manuel, OE Bamigbowu, GE Ossai
Delta State University Teaching Hospital, Oghara: E Umuerri**, P Oghojamoni-Ogefere, M Oyovwevotu
University of Abuja Teaching Hospital, Abuja: DB Ojji**, OB Ajanya, C Alo-Joseph, O Daniel, AE Ibrahim, E Ngada, N Ripiye, F Taiwo, I Ubah
University of Ilorin Teaching Hospital, Ilorin: J Ogunmodede**, S Adeniyi, O Olugbola
Aminu Kano Teaching Hospital, Kano: MU Sani**, IM Alfa, T Abdussalam, UA Abdullahi
Usmanu Danfodiyo University Teaching Hospital, Sokoto: SA Isezuo**, MA Zagga, H Umar, HE Okocha, NP Aloja
University of Maiduguri Teaching Hospital, Maiduguri: F Buba**, MA Talle, NA Ahmadu, IA Galtimari, AA Tukur, Z Zakariyya
Federal Teaching Hospital Gombe : YA Ayoola**, RA Oyekunle, S Adamu

Tanzania
Tanzania Cardiac Society, R Mvungi
A Makubi*
Jakaya Kikwete Cardiac Centre, Dar es Salaam: A Makubi**, P Kisenge, S Msigwa
Benjamin Mkapa Hospital, Dodoma: J Meda**, N Magitta, A Chandika

Europe
Poland
Polish Cardiac Society, P Mitkowski
P Jankowski*
Department of Population Medicine and Lifestyle Diseases Prevention, Medical University of Białystok, Białystok: K Kamiński**, M Łapińska
Department of Cardiology Medical University of Białystok, Białystok: B Sobkowicz**, M Knapp, K Mickiewicz
Department of Invasive Cardiology Medical University of Białystok, Białystok: S Dobrzycki**

Department of Cardiosurgery Medical University of Białystok, Białystok: T Hirnle**, K Charkiewicz-Szeremeta
Department of Cardiology, Białystok Regional Hospital, Białystok: J Bychowski**, A Guzowska – Suchowolec
Department of Cardiac Rehabilitation and Health Promotion, Collegium Medicum Nicolaus Copernicus University in Toruń, Toruń: A Kubica**, P Michalski, A Kosobucka-Ozdoba, Ł Pietrzykowski, A Rzepka-Cholasińska, J Ratajczak, M Siedlaczek
Clinical Department of Cardiology and Intensive Cardiac Therapy L. Rydygier in Toruń, Toruń: G Skonieczny**, P Kostrzewa
2nd Department of Cardiology, Upper-Silesian Medical Centre, Medical University of Silesia, Katowice: Z Gąsior**, Ł Maciejowski, M Matla-Hajzyk, B Hapeta-Zeman, K Szóstak-Janiak
Department of Cardiology and Hypertension, Central Research Hospital, the Ministry of the Interior and Administration, Warsaw: D Kosior**, P Kulak, E Rajska,
Center of Cardiology, American Heart of Poland Group, Jozefów: D Kosior**, A Rak
Department of Internal Medicine and Geriatric Cardiology, School of Public Health, Centre of Postgraduate Medical Education, Warsaw: P Jankowski**, D Koczwarska-Maciejek
Department of Nursing Management and Epidemiology Nursing, Institute of Nursing and Midwifery, Faculty of Health Sciences, Jagiellonian University Medical College, Krakow: R Wolfshaut-Wolak**

Portugal

Portuguese Society of Cardiology, L Gonçalves, H Pereira
A Abreu*
Hospital São João, Porto: M Carvalho**, A Amador, J Calvão, C Costa, H Moreira, P Palma, R Pinto, T Proença, M Rocha
Hospitalar e Universitário de Coimbra, Coimbra: N Moreira**, J Almeida, G Batista, J Borges-Rosa, G Campos, C Fernandes, J Guimarães, S Martinho, T Santos, A Silva, M Simões
Centro Hospitalar de Leiria, Leiria: J Morais**, M Carvalho, C Gonçalves, A Martins, A Vazão
Centro Hospitalar Universitário de Lisboa Norte, Lisbon: A Abreu**, P Alves da Silva, J Brito, M Lemos Pires, AM Lima Martins, F Pinto, R Pinto
Centro Hospitalar Lisboa Ocidental, Lisbon: C Aguiar**, R Amador, R Bello, R Lima
Hospital Espírito Santo de Évora, Évora: J Pais**, A Almeida, M Caria, M Carrington, D Conde, C Francisco, K Kongo, L Patricio, M Peralta, E Rebola, R Rocha, F Silva, M Trinca
